# Pedestrian Detection in Blind Area and Motion Classification Based on Rush-Out Risk Using Micro-Doppler Radar †

**DOI:** 10.3390/s21103388

**Published:** 2021-05-13

**Authors:** Sora Hayashi, Kenshi Saho, Daiki Isobe, Masao Masugi

**Affiliations:** 1 Department of Electronic and Computer Engineering, Ritsumeikan University, Shiga 525-8577, Japan; ri0063ir@ed.ritsumei.ac.jp (S.H.); ri0077vk@ed.ritsumei.ac.jp (D.I.); masugi@fc.ritsumei.ac.jp (M.M.); 2 Department of Intelligent Robotics, Toyama Prefectural University, Toyama 939-0308, Japan

**Keywords:** motion classification, blind area, micro-Doppler radar, prediction of rush-out

## Abstract

Various remote sensing technologies have been applied in intelligent vehicles and robots for surrounding-environment recognition. However, these technologies experience difficulties in detecting pedestrians in blind areas and their motions, such as rush-out behaviors. To address this issue, we present a radar-based technique for the detection of pedestrians in blind areas and the classification of different risks of rush-out behaviors among detected pedestrians. We verify their ability to detect pedestrian motion in blind areas by conducting experiments in two environments with blind areas formed by outdoor cars and indoor walls. Then, the classification of motions with different risks of rush-out behaviors among pedestrians detected in the blind areas is demonstrated. We use the clustering method to accurately classify several types of behaviors with different rush-out risks in both environments.

## 1. Introduction

Sensing technology for detecting the surrounding environment using various types of remote sensors, including cameras, radars, and lidars, has become ubiquitous. Such technologies have been implemented in various intelligent vehicle applications, such as advanced driver-assistance systems [1,2] and the autonomous driving of cars [3], and in various robots, such as agricultural robots [4,5] and indoor robots [6]. However, the current problem with sensing technology for the recognition of the surrounding environment is its difficulty in detecting people and objects in blind areas. Blind areas are regions, such as behind a wall or between vehicles, that cannot be directly detected by a measurement sensor. Monitoring the blind area is crucial for the applications mentioned above because pedestrians’ rush-out from blind areas often results in serious accidents that may cause serious injury to pedestrians.

For detecting pedestrians in blind areas, methods using mutual communication between vehicles (known as the V2V communication) have been proposed [7]. This method serves as an example of the monitoring of blind areas as it pertains to advanced driver-assistance systems. In this method, one vehicle can only detect pedestrians in blind areas when the other vehicle can detect the same. Therefore, this method is not versatile because it requires two or more vehicles to detect pedestrians in blind areas. As another similar approach, methods for detecting pedestrians via mutual communication between a pedestrian’s communication device and a vehicle (known as the V2P communication) have been proposed [8]. However, this method cannot be used to detect pedestrians who do not have a communication device.

Another approach for addressing the issues of pedestrian recognition in blind areas is a remote sensing-based scheme using cameras and lidars [9]. Because these sensors can recognize different measurement areas, the sensor fusion approach to detect pedestrians in the blind area (occluded pedestrians) has been studied [10]. However, these techniques assumed that the pedestrians are partially visible and almost all studies do not assume the pedestrians that are completely invisible. Furthermore, these conventional studies mainly focus on the detection and recognition of target types (e.g., human or not) and the consideration of rush-out risk is not assumed to the authors’ best knowledge. To address the problems mentioned above, radar sensing can be used to detect humans and objects in blind areas owing to the propagation characteristics of radio waves, including diffraction and multiple reflections. For example, Fujita et al. and Johansson et al. [11,12] proposed a radar imaging method that uses multiple reflected waves from walls. Such methods use reflections from multiple walls to detect objects in blind areas that are invisible to the radar installed indoors. Additionally, Zhang et al. [13] proposed a radar imaging technique using a diffraction phenomenon to detect an object behind a wall through which radio waves cannot pass. However, in these studies, experiments were performed in an anechoic chamber or in an environment surrounded by omnidirectional walls and through simulation-based investigations. Furthermore, there are only a few studies on the detection of pedestrians in blind areas. Bartsch et al. [14] investigated the detection of pedestrians occluded by cars based on a range-Doppler map. However, the accuracy of their method was not fully verified, and it aimed to classify pedestrians or static objects. Furthermore, the effects on radio propagation are not discussed and its applicability to the completely invisible blind area composed by the walls is not confirmed, similar to the previous studies that used cameras and lidars Additionally, the detection of rush-out behaviors was not considered. He et al. [15] recently presented experimental results on the classification of objects in blind areas. However, this study showed only the classification of humans and metallic cylinders through experiments in a laboratory setting. Therefore, there are no reports on the investigation of radar-based detection of the movements of pedestrians in blind areas, including rush-out behaviors in a realistic environment.

In this paper, we propose a radar-based method for classifying the rush-out risks of pedestrians in blind areas by utilizing the characteristics of radio waves, such as multiple reflections and diffraction phenomena. We verify the effectiveness of the proposed method by conducting experiments in both outdoor and indoor environments. In the outdoor environment, a car was used to form a blind area, and in the indoor environment, walls were used to form a blind area that completely occludes pedestrians. First, to demonstrate the possibility of monitoring the motions of pedestrians in blind areas, we revealed the ability to detect pedestrian participants located in a blind area using a micro-Doppler radar. We then applied the measured data to the short-time Fourier transform to obtain a spectrogram (time–velocity distribution) that reflected the gait characteristics. We extracted features from the spectrogram and classified them using Ward’s clustering method [16], which is an unsupervised machine learning technique. Through our proposed method, we classified walking motions with different risks of rush-out behaviors. The contributions of this study are summarized as follows.

- The radar-based detection of rush-out risk of pedestrians is experimentally investigated for the first time.
- For the radar-based blind area monitoring, the recognition of the motions related to the behaviors of the rush-out risks has been achieved in realistic situations whereas the conventional studies considered the detection or simple classification problem for the detected target (e.g., classification of human or not).
- The measurements of the completely occluded pedestrians are considered whereas the conventional studies using the cameras and lidars considered the partially occluded humans.
- The quantitative data on the detection capability of the humans in blind areas using the radar are provided and the radio propagations related to the detection mechanism are discussed using the results in the realistic indoor and outdoor environments.
- We show that the simple clustering method based on the motion feature parameters extracted from the radar spectrograms yielded accurate classifications of motions related to the rush-out risks. The feature parameters that can be considered as the related parameters of rush-out risks were extracted.

Note that this study is an extended version of our conference paper [17]. In this study, we add the results of experiments conducted in an outdoor environment in which we assume that a blind area is formed by a car, and we add related discussions considering the experiments for both indoor and outdoor environments.

## 2. Experimental Environments and Radar Specification

**Figure 1:**
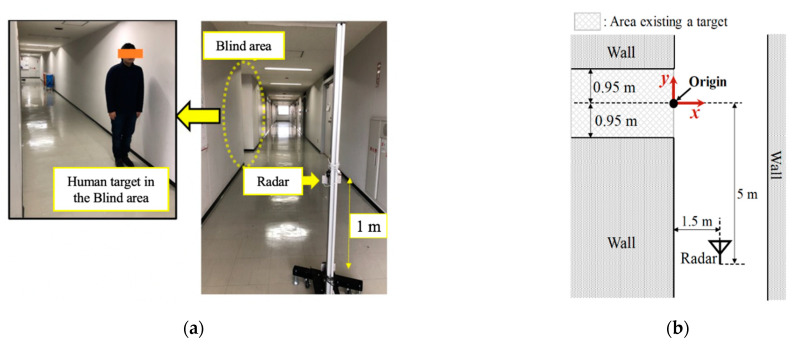
Experimental site and measurement system model for the indoor environment. (**a**) Experimental site; (**b**) Measurement system model.

**Figure 2:**
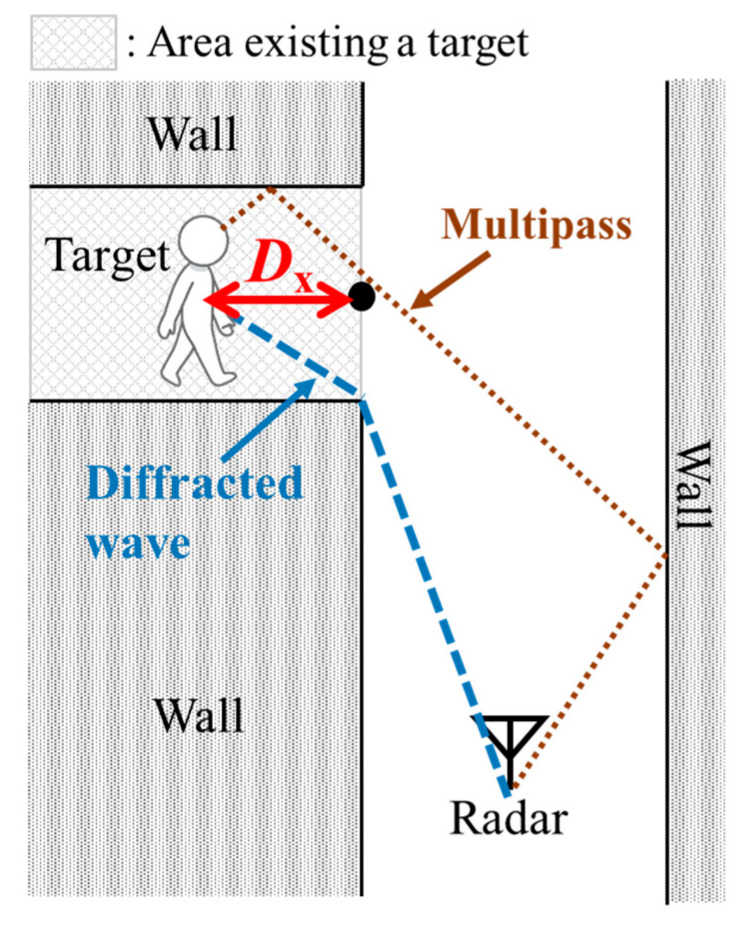
Main radar radio propagation paths in the indoor experiments.

We conducted experiments in two types of realistic environments: indoor and outdoor. Figure 1 depicts the experimental site and a radar measurement system model used for the former. The micro-Doppler radar was installed at (*x*, *y*) = (1.5 m, −5.0 m) at a height of 1.0 m. It emitted electromagnetic sinusoidal waves with a frequency of 24 GHz. The antennas had directivities of ±35° and ±14° for the H-plane and E-plane, respectively, and an effective isotropic radiated power for transmitting waves of 40 mW. The received signals were demodulated by the transmitted signals and were composed of Doppler frequencies corresponding to the walking velocities of pedestrians. The sampling frequency of the received signal was 600 Hz, which corresponded to a maximum measurement velocity of 3.75 m/s. A human target was placed in the middle of an aisle behind a wall, which was not directly visible using the radar. In this study, this invisible area directly from the radar is called the blind area. Figure 2 illustrates the main radar radio propagation paths in the indoor experiments. The received signals are mainly obtained as diffracted waves from the edge of the corner and double reflected waves from the walls. For both environments, we defined the absolute value in the x-axis of the participant position as *D*_x_. When *D*_x_ was large, the participant was in relatively deep in the blind area.

**Figure 3:**
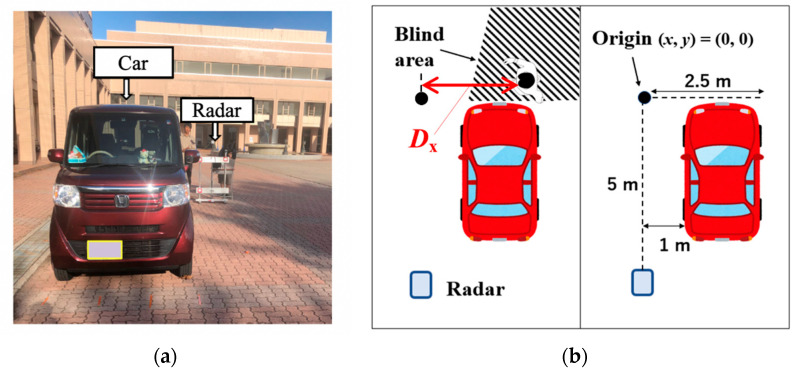
Experimental site and measurement system model for the outdoor environment. (**a**) Experimental site; (**b**) Radar measurement system model.

We also performed experiments for an outdoor environment in which the blind area was formed using a car. Figure 3 depicts the experimental site and radar measurement system model used in the outdoor environment. There were no reflective objects, such as walls, around the vehicle. Therefore, multipath from the surrounding environment could not be obtained. The same radar used in the experiments for the indoor environment was used in the outdoor environment. The radar was installed 1 m away from the vehicle along the x-axis. In Figure 3, radar heights were set to 0.1 m. The aim of the height of 0.1 m was to propagate radio waves in the lower space between the car and ground. The subjects walked close to the car. The car used in the experiment was N-BOX, which is manufactured by Honda Motor Co., Ltd. (Tokyo, Japan). The total height, width, and overall length of this car are 1.8 m, 1.475 m, and 3.495 m, respectively.

## 3. Methods for Evaluating Detection Capability and Motion Classification

Based on the signals received by the radar, we demonstrated the ability to detect human movements in a blind area. We assumed the detection of the participants using walking movements and aimed to detect the components corresponding to their motions using spectrograms (time–velocity distribution) of the received signals. The spectrograms were calculated using the short-time Fourier transform [18], wherein a hamming-window function with a length of 128 samples and an overlap length of 127 samples were used. We investigated the mean power density of the components corresponding to the walking motion in the spectrogram for several participant position Dx values to evaluate their detection capability in the blind area.

Then, the movements of the participants detected in the blind area were classified according to the risk of rush-out behaviors using the clustering method. Similar to our previous study [18], the gait velocity parameters were extracted from the spectrograms. We extracted their envelopes, which corresponded to the maximum velocity, minimum velocity, and maximum power at each time. In calculating these envelopes, we detected the received power above a threshold level set to >1 dB/Hz. Next, we extracted the velocity parameters from the spectrogram, namely, *v*_mean_, *v*_u,mean_, and *v*_l,mean_. These are defined as the mean of the maximum power components, the maximum velocity envelope, and the minimum velocity envelope with respect to time, respectively. We also use the difference between *v*_u,mean_ and *v*_l,mean,_ which is defined as Δ*v* = (*v*_u,mean_ + *v*_l,mean,_)/2 for the feature parameter. These feature parameters are ideal for detecting the rush-out risks because the *v*_mean_, *v*_u,mean_, and *v*_l,mean_ indicate the offset value of the velocities and these values indicate the difference of the walking direction and Δ*v* corresponds to the degree of variation of leg velocities whose value becomes large when the participant moving with relatively large leg motions. When *v*_mean_, *v*_u,mean_, and *v*_l,mean_ are larger values and possess a large |Δ*v*|*,* the possibility of the high rush-out risk becomes higher.

The movement types of the participants in the blind area were classified using Ward’s clustering method [16] using the extracted velocity parameters. The pedestrian movements were classified into the following walking motion types with different risks of rush-out behaviors.

- Walking from the blind area to the visible area (BtoV type): This walking motion type is assumed to have a high probability of rush-out behaviors from the blind area.
- Walking from the visible area to the blind area (VtoB type): It is assumed that in this walking motion, the human is unlikely to rush-out behaviors from the blind area.
- Walking in place in the blind area (WiP type): This walking motion type is assumed to have a relatively large motion with a low probability of rush-out behaviors from the blind area.

## 4. Results and Discussion

### 4.1. Results for Indoor Environment

**Figure 4:**
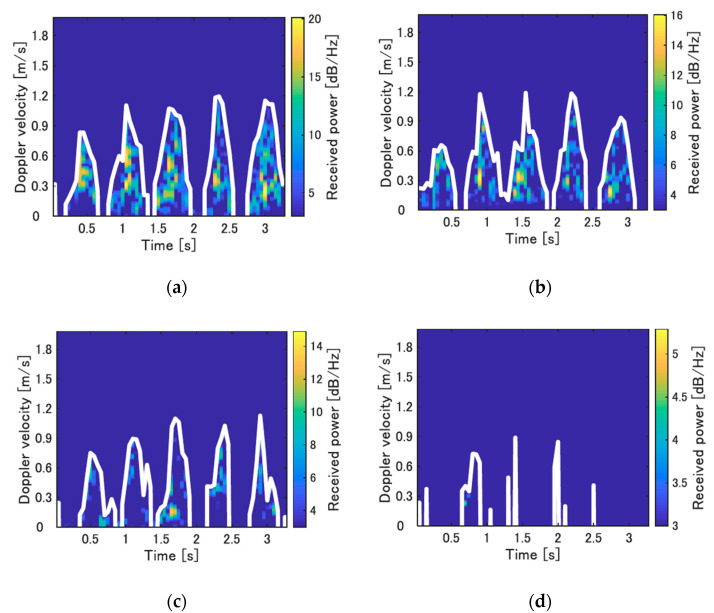
Spectrograms of the human subject who walked in place for each *Dx* and their maximum velocity envelope (white solid line) (indoor environment). (**a**) *D*_x_ = 0.5 m; (**b**) *D*_x_ = 0.7 m; (**c**) *D*_x_ = 0.9 m; (**d**) *D*_x_ = 1.1 m.

**Figure 5:**
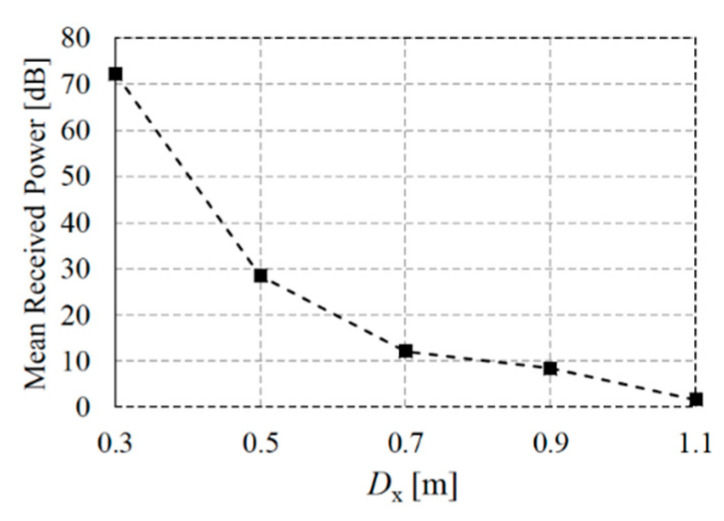
Mean received power corresponding to the significant peaks in the spectrogram for each *D*_x_ (indoor environment).

We first demonstrate the ability of the system to detect the human walking movements in the blind area behind the wall. The experiment was conducted in an indoor environment. The experimental participant was a man aged 22 years with a height of 173 cm. He walked at the position *(x, y)* = (−*D*_x_, 0.55 m). The mean of the received power of the components in the spectrogram corresponding to the reflected waves from the subject was calculated for several *D*_x_ values. Figure 4 depicts the spectrograms for *D*_x_ *=* 0.5 m, 0.7 m, 0.9 m, and 1.1 m, and the maximum velocity envelopes that correspond to the leg swinging while walking. When *D*_x_ is less than 0.9 m, the periodic components corresponding to the subject walking can be confirmed. The subject at *D*_x_ = 0.5 m exists in the blind area, and thus, it is possible to detect the subject’s movements using the diffracted waves and multiple reflected waves. However, the mean received power is significantly reduced for *D*_x_ = 1.1 m, which can make the recognition of the movements using measured data difficult. Therefore, it is difficult to estimate the periodic walking component of the subject at *D*x = 1.1 m in this environment. Figure 5 depicts the results of the mean received power corresponding to the significant peaks in the spectrogram for each *D*_x_. The received power is attenuated to approximately 2% between *D*_x_ = 0.3 m and *D*_x_ = 1.1 m, which implies that the detection capability is significantly reduced for larger *Dx.*

After the human is detected in the blind area, we then classify movements that correspond to different possibilities of rush-out from the blind area. For the three assumed motion types, the motions of the participants were as follows:

- BtoV type: The subject walked from (*x*, *y*) = (−2 m, 0 m) in the positive direction of the x-axis. The received signal corresponding to x1 m < *x* < −0.2 m was used for the classification
- VtoB type: The subject walked from the origin in the negative direction of the x-axis. Similar to the BtoV type, the received signal corresponding to −1 m < *x* < −0.2 m was used for this classification.
- WiP type: The participant stamps in (*x, y*) = (−0.5, 0 m). The length of the received signal used for this classification was the same as that of the BtoV type.

**Figure 6:**
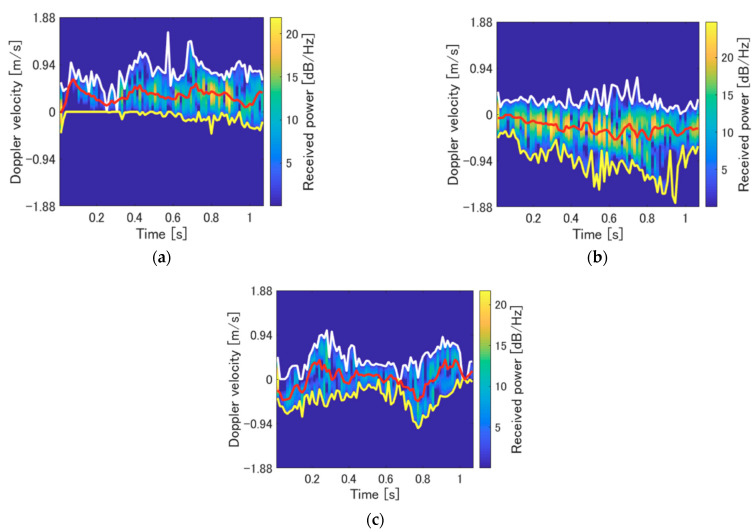
Representative spectrograms for each motion type (indoor environment). The white, yellow, and red lines correspond to maximum velocity, minimum velocity, and maximum power components for each time, respectively. (**a**) blind area to the visible area (BtoV) type; (**b**) visible area to the blind area (VtoB) type; (**c**) walking in place in the blind area (WiP) type.

The experimental participants were 10 young men (21–24 years old, with a mean height of 176.2 cm). Each of the subjects performed the three walking motion types mentioned above five times. Therefore, the number of data points used for classification was 50 for each motion type. Figure 6 depicts the representative spectrograms for each walking motion type. As shown in these figures, the differences in the walking direction of the movements of the BtoV and VtoB types were obtained as the bias of the velocity components. Although the movements of these types were in a lateral direction to the radar (these are hard-to-detect directions for the Doppler radars), their velocity components were clearly confirmed through the detection of multiple reflections of scattering centers on the legs. As shown in Figure 6c, the relatively large motion of the legs of the WiP type was clearly confirmed. There is no bias in the velocity components in the WiP type, which indicates that the subject has a high motion speed but remains in place.

**Figure 7:**
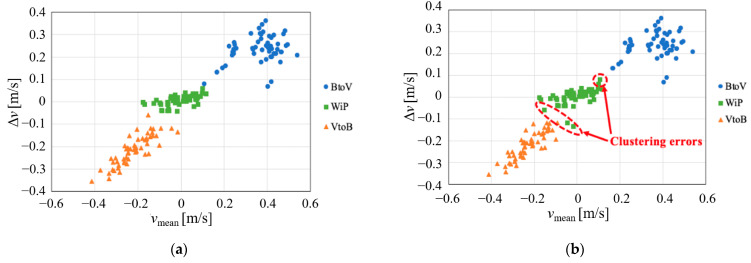
Results of velocity parameter estimation and clustering (indoor environment). (**a**) Velocity parameters for true motion types; (**b**) Clustering results.

Figure 7 depicts the classification results using Ward’s clustering method, whose feature parameters are *v*_mean_ and Δ*v*. Clearly, there were significant differences in each movement type. The difference between the maximum and minimum velocity components emphasized the small difference in the WiP type between the legs, making it easy to distinguish them from the other two types. In the other two types, the difference between the positive and negative of the bias of the velocity component appears with both parameters, making it easy to distinguish them. Thus, the clustering results depicted in Figure 7b indicate that there were only four misclusters, which corresponded to a clustering rate of 97.5%.

### 4.2. Results for Outdoor Environment

**Figure 8:**
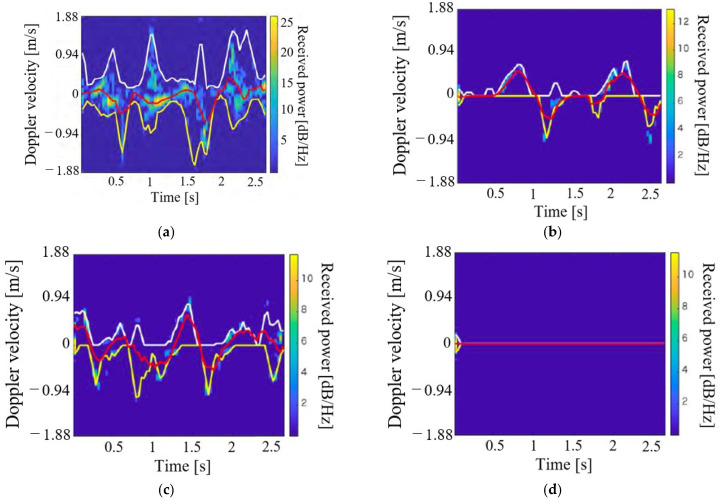
Spectrograms of the human target that walked in place for each *D*_x_ (outdoor environment). (**a**) *D*_x_ = 1.0 m; (**b**) *D*_x_ = 1.5 m; (**c**) *D*_x_ = 2.0 m; (**d**) *D*_x_ = 2.5 m.

**Figure 9:**
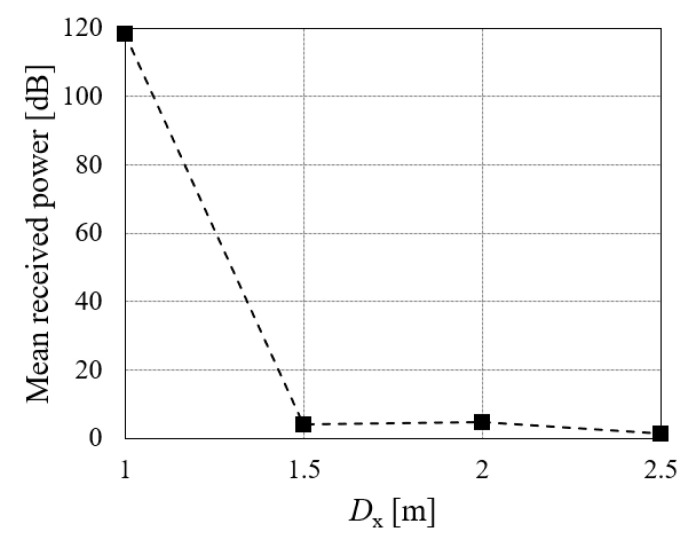
Mean received power corresponding to the significant peaks in the spectrogram for each *D*_x_ (outdoor environment).

Similar to the experiments for the indoor environment, we first investigated whether pedestrians in the blind area formed by the vehicle could be detected with sufficient received power. The subject was a man aged 23 years of height of 169 cm. He stamped in (*x, y*) = (*D*_x_, 0 m). The mean of the received power of the components in the spectrogram corresponding to the reflected waves from the subject was calculated for several *D*_x_ values. Figure 8 depicts the spectrograms for *D*_x_ = 1.0 m, 1.5 m, 2.0 m, and 2.5 m. As shown in Figure 8, the periodic components of the walking motion can be confirmed when *D*_x_ = 1.0 m, 1.5 m, and 2.0 m. In contrast, it is difficult to detect walking motion at *D*_x_ = 2.5 m because the received power is significantly reduced. In terms of the radar, the subject’s foot at *D*_x_ = 2.5 m overlaps the vehicle’s right front wheel. Therefore, it is considered difficult to detect the subject because the radio waves do not propagate directly. When the subject at *D*x = 1.0 m was stationary, most of the subject was not directly visible from the radar. However, when the subject performed a walking motion there, his hands and feet were directly visible. On the other hand, when the subject with *D*_x_ = 1.5 m performed a walking motion, the subject’s toes were visible through the lower space of the vehicle. Therefore, there was a large difference in spectrogram received power between *D*_x_ = 1.0 m and 1.5 m. Figure 9 depicts the results of the mean received power corresponding to the significant peaks in the spectrogram for each *D*_x_. The received power is strongly attenuated for *D*_x_ = 1.5–2.5 m compared with that for *D*_x_ = 1.0 m. The mean received powers for *D*_x_ = 1.5 and 2.0 m were approximately 4 dB, which implies the smaller detection capability. However, as indicated in the spectrograms, some features of the motions can be confirmed in these cases. In contrast, the received power for *D*_x_ = 2.5 m was approximately 1 dB and the detection of the subject was difficult. From the above results, it is possible to detect the pedestrian in the blind area using radar by propagating radio waves to the space under the vehicle and measuring the walking motion of the subject’s toes including the smaller received power cases for *D*_x_ = 1.5 and 2.0 m.

**Figure 10:**
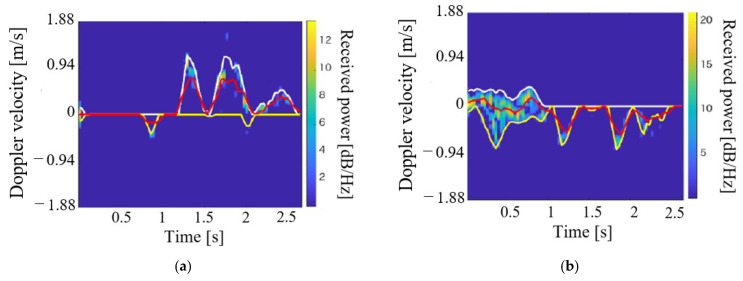
Examples of spectrograms for each walking motion type (outdoor environment). (**a**) BtoV type; (**b**) VtoB type.

**Figure 11:**
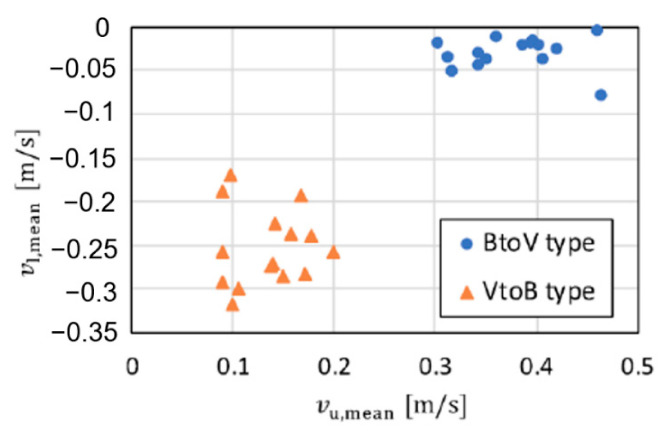
Estimated velocity parameters for each motion type (outdoor environment).

Next, we demonstrate the clustering of motions with different rush-out risks in the outdoor environment. For simplicity, we assume the classification of the BtoV and VtoB types. The experimental participants were three healthy young men (21–23 years old, with a mean height of 170.0 cm). The participants performed the two motion types five times each. Therefore, the numbers of data for the clustering were 15 for each motion type. Figure 10 depicts the representative spectrograms for each walking motion type. The leg movements during walking are illustrated as abrupt velocity changes in the spectrogram. From the BtoV type spectrogram, it is possible to measure the walking motion for approximately three steps immediately before rushing out. In contrast, the VtoB type has a toe at the start of walking (1.0 m, 0 m), as shown in Figure 3b. Therefore, the velocity component of one step, including the received signal of the entire subject’s body from the start of measurement to approximately 1.0 s and approximately three steps of walking in the blind area can be measured from the spectrogram. Figure 11 depicts the clustering results using the extracted parameters *v*_u,mean_ and *v*_l,mean_. Clearly, the accuracy was 100% for the classification of the two motion types owing to their clear divergence in the maximum and minimum envelopes, as indicated in Figure 10.

### 4.3. Overall Discussion

The results of the experiments in both indoor and outdoor experiments demonstrate the ability of the proposed method to detect pedestrians in blind areas and classify walking patterns according to high or low risks of rush-out behavior among the detected subjects. Thus, the 24 GHz micro-Doppler radar is efficient in terms of monitoring human targets in various blind areas based on the propagation of radio waves, such as multiple reflections and diffraction. The main contributions of our study are as follows:

- We investigated the detectable distance from the visible area for the participants in the blind area in realistic situations: the indoor environment assumed realistic measurement situations for indoor robots and the outdoor environment assumed realistic situations for various vehicles, including cars, motorcycles, bicycles, and other vehicles.
- We classified the motions of participants with different rush-out risks using a clustering method and simple motion parameters extracted from the spectrograms. This is clearly more effective than the conventional method proposed in the study reported in [15], which classified the target detected in a blind area as a human or metallic cylinder.

According to the results of the investigation on the ability to detect the participants behind the wall in the indoor environment, the attenuations of the received power in the blind area were remarkable, and the detection of the subject located at *D*_x_ = 1 m or more was relatively difficult. However, these results also indicated that the detection of the participants and the recognition of their movements were possible in areas where direct waves did not propagate owing to the wall as well as multipath and diffraction. However, the received power decreased significantly as the distance from the visible area decreased, and this was affected by the radar transmission frequency. For example, although our Doppler radar used 24 GHz waves, the effects of the diffraction were relatively large when we used radio waves with smaller frequencies, such as the 2.4 GHz band. Therefore, there is a possibility that at a long distance, they can be detected in such low frequencies. This is an important task for future studies. However, in other words, the important finding of our study was that the Doppler radar, even when using the relatively larger frequency of 24 GHz, can detect moving humans in the blind area and the risks of their rush-out behavior to some extent.

Additionally, it is assumed that the direct waves and the waves reflected by the ground, which are propagated under the car, reached the pedestrian participants in the blind area for the outdoor environment experiment, and their effects on diffraction were fewer. Therefore, pedestrian detection may be more difficult than demonstrated in our experiments when using a car with a low clearance from the ground. In addition, some situations can use echoes of multipath from other vehicles, walls of buildings, and/or guardrails, even for outdoor environments, similar to the indoor environment. Such situations can detect pedestrians in blind areas with higher sensitivity.

In summary, the results of this experiment vary depending on the conditions of the environment. Therefore, the considerations of only the blind areas formed by the wall and car are the limitations of our study. However, this study is the first to experimentally demonstrate the possibility of detecting pedestrians in blind areas and their rush-out risks in realistic situations by assuming both indoor and outdoor environments that can use the effects on diffractions and multipath echoes.

### 4.4. Contribution and Limitation of This Study

We now discuss the contributions of our study. The contribution of this study is the demonstration of the effectiveness of the microwave radar for monitoring the blind area assuming the detection of the realistic rush-out situation for the first time. Because we used the microwave radar, our method can seamlessly operate under rainy or snowy conditions [19], fogs and smokes [20], and blind areas that are occluded by other obstacles such as plants [21]. Furthermore, our indoor measurements in this study assumed completely occluded pedestrians by the wall, which is not assumed in the previous study on the rush-out detection. Thus, our results revealed the application capability of the radar-based technique for such situations. In addition, our technique can be applied to the detection of the rush-out behaviors of pedestrians that are not in blind areas. The experimental studies on the detection of the rush-out behavior, including the assumption of non-blind area, have not been widely reported. For example, many studies on V2V and V2P communications are based on simulations or experiments, and they do not consider rush-out detection. Although an experimental study on the rush-out risk using smartphones and accelerometers was recently studied [22,23], such experimental studies that used remote sensing techniques have not been reported. Thus, our study experimentally shows the potential for detecting rush-out behaviors in the realistic environment using the radar for the first time and its applicability to pedestrians in blind areas.

However, this study had two main limitations. The first is that we measured the motion of a single pedestrian, and the situations for several pedestrians in the measurement area are not considered. However, the Doppler radar can differentiate multiple targets based on the differences of the velocities of detected targets and the separation based on control of the beam illumination area is also an efficient method [24]. Using these methods, we can separately detect several pedestrians, and additional experiments are required for conditions with several pedestrians. Second, we set radar parameters in order to measure the walking motion velocities, and other important targets such as cyclists and motorcyclists were not considered. The limitation of the measured velocity depends on the frequency of the transmitting signals and the sampling interval of the received signals: the lower transmitting frequency and higher sampling intervals spread the measurement range of the Doppler velocities. The maximum measurement velocity of our radar setting was 3.75 m/s. Although this value is sufficient for measuring the walking motion (the walking velocity of humans is approximately 0.7–1.9 m/s [25]), it is not suitable for cyclists and motorcyclists. Moreover, when we integrate the radars in fast vehicles (such as cars), the relative speed of the pedestrian from the vehicles becomes large and the measurement range of the velocity should be also large. However, because we can use smaller transmitting frequencies such as 2.4 GHz and smaller sampling frequencies, the measurements of such fast targets will be realized: this investigation will be a part of the future work.

## 5. Conclusions

In this study, we performed experiments with the aim of predicting the rush-out behaviors of humans from a blind area for the first time. The experiments for an indoor environment, whose blind area is formed using a wall that completely occluded the target pedestrians, investigated the ability to detect a moving human target in a blind area and the classification performance of the three movements with different possibilities of rush-out behavior. The information on the moving human target behind the corner was obtained from the received signals through the diffraction and multiple reflections of the radio waves. In our results, a target existing in the blind area approximately 1 m away from the visible area was detectable. Furthermore, a classification accuracy of 97.5% was achieved for the three motion types, which indicates the possibility of detecting the rush-out from a blind area using Ward’s clustering method of the velocity parameters obtained using the micro-Doppler radar. The experiments conducted in the outdoor environment investigated the ability to detect a moving human target in a blind area and the classification performance of the two movements with different possibilities of rush-out behavior. We measured the walking motion of the subjects behind the car by propagating radio waves in the lower space of the car. The classification of the risks of rush-out behavior was performed completely. That is, we revealed that the simple clustering method using the spectrogram features can achieve accurate detection of the rush-out behavior; which is the notable result for the radar remote sensing technology even if the pedestrian is not occluded by obstacles. These results indicate the potential of the Doppler radar for use in the detection of humans with high-rush-out risk in the blind area including the completely occurred situations. They have yielded collision avoidance systems that can be used in intelligent transportation systems and moving robots to protect pedestrians from accidents.

However, future experiments are needed to resolve the limitations described in the previous section. In addition, the results of the experiments depend on the radio wave propagation environment and transmission signal power, among other factors. Therefore, in our future studies, we shall perform other experiments for various parameters related to these conditions, particularly the use of other radio frequencies, such as the 2.4 GHz band.

## Data Availability

The data presented in this study are available on request from the corresponding author.
